# Using performance-based financing (PBF) to motivate health commodity supply chain improvement at a central medical store in Mozambique

**DOI:** 10.1186/1472-6963-14-S2-P148

**Published:** 2014-07-07

**Authors:** Brian Serumaga, Cary Spisak, James Rosen, Lindsay Morgan, Rena Eichler

**Affiliations:** 1 John Snow Inc., Arlington, Virginia, USA; 2 Broad Branch Associates, Washington, DC, USA

## Background

The predominant model of public health commodity supply chains in developing countries is one dominated by a central medical store (CMS). In this model, the CMS plays the pivotal role of procurement, storage and warehousing of all health commodities before they are distributed to the next level in the supply chain. Challenges with technical and organization capacity at the CMS level has led to longstanding difficulties in creating sustainable performance improvements in several countries. In Mozambique, the central medical store (Central de Medicamentos e Artigos Médicos-CMAM) receives significant US government support (through USAID) for both health commodities and technical assistance. We tested the effectiveness of a PBF scheme between CMAM and USAID, to improve the functioning of the CMS in Mozambique.

## Materials and methods

In January 2013, USAID entered into a year-long government to government grant arrangement that conditions disbursement of tranches of USAID support on specific results at CMAM. The disbursements would take the form of a fixed amount reimbursement award (FARA) of up to $125,000 per quarter ($500,000 per year) if CMAM could demonstrate meeting quarterly targets on six performance indicators. These indicators were related to planning, distribution, and warehouse management. The aim of the PBF program was to spur innovation, hard work and improve warehousing.

We hypothesized that the incentive would lead to improvements through three pathways:

1. Improved staff motivation and morale due to individual or group bonus payments

2. Improved collaboration between and within CMAM departments due to the need for cooperation among departments in order to achieve the performance targets, and

3. Increased targeted investments in infrastructure, systems and human resources, due to the additional funds available to CMAM through the grant.

Indicators were selected in areas where change had previously been difficult to achieve, where baseline data could be collected, where performance was entirely under CMAM's control, and for which measurable targets could be set and achieved within 1 year of the program. Baseline data was collected in the last quarter of 2012.

## Results and conclusions

We found improvements in all indicators over one year. Matching records of stock status reports and physical counts improved from 70% at baseline to over 85% by 2013. There were improvements in picking accuracy, order cycle times and distribution planning. The incentive led to better collaboration between CMAM departments.

We found process improvements due to the PBF scheme, possibly leading to increased availability of health commodities.

